# Point Mutations in FimH Adhesin of Crohn's Disease-Associated Adherent-Invasive *Escherichia coli* Enhance Intestinal Inflammatory Response

**DOI:** 10.1371/journal.ppat.1003141

**Published:** 2013-01-24

**Authors:** Nicolas Dreux, Jérémy Denizot, Margarita Martinez-Medina, Alexander Mellmann, Maria Billig, Dagmara Kisiela, Sujay Chattopadhyay, Evgeni Sokurenko, Christel Neut, Corinne Gower-Rousseau, Jean-Frédéric Colombel, Richard Bonnet, Arlette Darfeuille-Michaud, Nicolas Barnich

**Affiliations:** 1 M2iSH, UMR1071 Inserm, Université d'Auvergne, USC-INRA 2018, Clermont-Ferrand, France; 2 Institute of Hygiene, University Hospital Münster, Münster, Germany; 3 University of Washington School of Medicine, Department of Microbiology, Seattle, Washington, United States of America; 4 Inserm U995, Université Lille II, Hôpital Claude Huriez, Lille, France; 5 Service de Bactériologie, CHU, Clermont-Ferrand, France; 6 Institut Universitaire de Technologie, Génie Biologique, Aubière, France; University of Utah, United States of America

## Abstract

Adherent-invasive *Escherichia coli* (AIEC) are abnormally predominant on Crohn's disease (CD) ileal mucosa. AIEC reference strain LF82 adheres to ileal enterocytes *via* the common type 1 pili adhesin FimH and recognizes CEACAM6 receptors abnormally expressed on CD ileal epithelial cells. The *fimH* genes of 45 AIEC and 47 non-AIEC strains were sequenced. The phylogenetic tree based on *fimH* DNA sequences indicated that AIEC strains predominantly express FimH with amino acid mutations of a recent evolutionary origin - a typical signature of pathoadaptive changes of bacterial pathogens. Point mutations in FimH, some of a unique AIEC-associated nature, confer AIEC bacteria a significantly higher ability to adhere to CEACAM-expressing T84 intestinal epithelial cells. Moreover, in the LF82 strain, the replacement of *fimH*_LF82_ (expressing FimH with an AIEC-associated mutation) with *fimH*_K12_ (expressing FimH of commensal *E. coli* K12) decreased the ability of bacteria to persist and to induce severe colitis and gut inflammation in infected CEABAC10 transgenic mice expressing human CEACAM receptors. Our results highlight a mechanism of AIEC virulence evolution that involves selection of amino acid mutations in the common bacterial traits, such as FimH protein, and leads to the development of chronic inflammatory bowel disease (IBD) in a genetically susceptible host. The analysis of *fimH* SNPs may be a useful method to predict the potential virulence of *E. coli* isolated from IBD patients for diagnostic or epidemiological studies and to identify new strategies for therapeutic intervention to block the interaction between AIEC and gut mucosa in the early stages of IBD.

## Author Summary

The etiology of inflammatory bowel diseases, in particular Crohn's disease (CD), involves disorders in host genetic factors and intestinal microbiota. Adherent-invasive *Escherichia coli* (AIEC) are receiving increasing attention because they have been reported worldwide to be more prevalent in CD patients than in healthy subjects. AIEC adhere to ileal enterocytes *via* type 1 pili, which recognize the CEACAM6 receptor, which is abnormally expressed in CD patients. The ability of AIEC to adhere to intestinal epithelial cells expressing CEACAM6 could be correlated with the presence of amino acid substitutions in the type 1 pili FimH adhesin subunit. AIEC strains express FimH protein variants with recently acquired amino acid mutations, which is a typical signature of pathoadaptive evolution of bacterial pathogens. AIEC-associated mutations in FimH confer on AIEC bacteria a significantly higher ability to adhere to CEACAM-expressing intestinal epithelial cells. Our results highlight a mechanism of AIEC pathogenic evolution that involves selection of FimH pathoadaptive mutations, which are required for AIEC gut colonization, which leads to the development of chronic inflammation in a genetically susceptible host. The analysis of *fimH* SNPs may be a useful method to predict the potential virulence of *E. coli* isolated from IBD patients in epidemiological studies and to develop new therapeutic interventions.

## Introduction

The molecular pathogenesis of inflammatory bowel disease (IBD), a chronic inflammation of the digestive tract, remains poorly understood. However, current evidence suggests that Crohn's disease (CD) pathogenesis involves interactions between the intestinal microbiome and the immune system, including important contributions from genetic and environmental risk factors with microorganisms playing a central role [1], [2]. Of the bacteria that may play a role in the pathogenesis of CD, a pathovar of *E. coli* called AIEC, for adherent-invasive *Escherichia coli*, has been strongly implicated in IBD, particularly in CD [3], [4], [5], [6], [7], [8]. AIEC are able to adhere to the intestinal epithelium and colonize gut mucosa. They invade intestinal epithelial cells and macrophages and are able to replicate intracellularly without inducing cell death or INFγ secretion by infected macrophages. AIEC were found to be associated with ileal mucosa in 36.4% of CD patients compared with 6.2% of controls [3], suggesting that these bacteria are involved in CD pathogenesis. AIEC are distinct from other pathogenic intestinal *E*. *coli* strains because they do not harbor genes typically associated with pathogens such as enterotoxigenic, enterohemorrhagic, enteroinvasive, enteroaggregative, and enteropathogenic *E*. *coli* [9], [10]. AIEC bacteria adhere specifically to carcinoembryonic antigen-related cell adhesion molecule 6 (CEACAM6), which is abnormally expressed in the ileal mucosa of 35% of CD patients, *via* FimH, the terminal subunit of the type 1 pilus [11].

Type 1 pili are encoded by the *fim* operon, and their expression is phase variable, depending on an invertible DNA element (the *fimS* region) that is located upstream of the *fim* operon and contains the *fim* promoter [12]. Two tyrosine recombinases, FimB and FimE, are known to control the orientation of the *fimS*-invertible region. FimB has bidirectional activity but predominantly switches *fim* operon transcription from OFF to ON, while FimE exclusively mediates ON to OFF phase switching [13], [14]. Additional FimB homologs also mediate type 1 pili phase variation *in vitro* and *in vivo* [15]. The FimH adhesin (approx. 32 kDa) consists of the lectin and the pilin domains, which are connected by a short tetrapeptide loop (residues 157–160), and is located on the tip of type 1 fimbriae, 0.5–1 µm long organelles primarily composed of FimA polymers found on the bacterial surface. FimH mediates bacterial adhesion in multiple body compartments including the large intestine, urinary tract, and bloodstream, in which the bacterium may be exposed to fluid flow. Through its lectin domain, the adhesin mediates bacterial binding to the ligand mannose, a carbohydrate found on host cells and surfaces in the form of monomannose (1 M) [16]. High monomannose binding depends on the presence of structural point mutations in the *fimH* gene [17]. FimH mutations were shown to confer significant advantages upon bacteria during bladder colonization in a murine model [18] and to correlate with extraintestinal virulence of *E. coli* [19]. In uropathogenic *E. coli* (UPEC), blocking the binding of FimH to its natural receptor prevents bacterial colonization and subsequent inflammation of the urinary tract [20], [21]. For example, mannosides, small molecule inhibitors of the type 1 pilus FimH adhesion, provide significant protection against catheter-associated UPEC urinary tract infections by preventing bacterial invasion and shifting the UPEC niche primarily to the extracellular environment [22]. Another strategy is to interrupt pilus assembly and thereby block pilus-mediated adhesion using pilicides, which are pilus inhibitors that target chaperone function by inhibiting pilus biogenesis [23], [24]. Finally, a vaccine containing a recombinant, truncated form of FimH adhesin strongly reduces *in vivo* bladder colonization by UPEC in a mouse cystitis model [25], and optimization of mannoside compounds for oral bioavailability shows therapeutic efficacy after oral administration and provides substantial benefit to women suffering from chronic and recurrent urinary tract infections [26].

Type 1 pili of AIEC bind with high affinity to overexpressed, mannosylated CEACAM6 in CD patients [11], and therapeutic strategies similar to those described for UPEC infection could be used to block gut colonization by AIEC in CD patients expressing CEACAM6. We therefore decided to investigate whether there is specific selection of the evolution of AIEC FimH adhesin. We elected to (1) analyze DNA variation patterns in *fimH* alleles among different AIEC and non-AIEC strains isolated from CD patients and controls, (2) correlate the presence of amino acid substitutions with the ability of *E. coli* strains to adhere to T84 intestinal epithelial cells expressing endogenous CEACAM molecules and (3) analyze the impact of AIEC *fimH* pathoadaptive polymorphism on the ability of AIEC to colonize and induce gut inflammation in an animal model expressing human CEACAMs.

## Results

### fimH allelic variation in AIEC and non-AIEC strains

**Figure 1:**
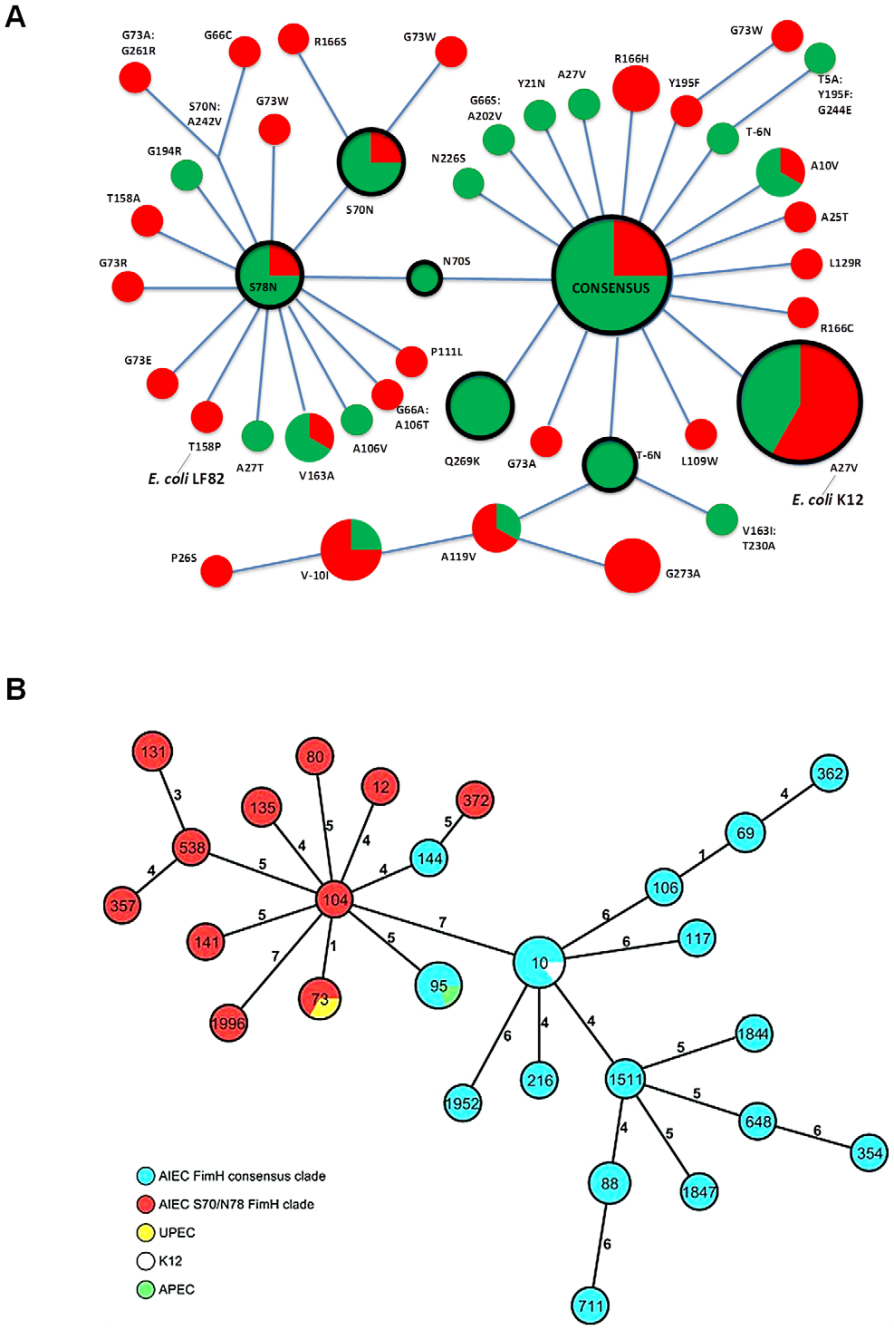
FimH protein Zonal Phylogeny and MLST tree of AIEC used in this study. (A) Each circle represents a FimH variant coded by phylogenetically distinct *fimH* allele(s). Circle size reflects the number of strains (from 1 to 12) carrying the corresponding FimH variant. Consensus, the most common and evolutionarily primary FimH variant. All amino acid changes indicated are derivatives from the consensus variant. Circles inside the black rings represent evolutionarily fixed FimH variants coded by multiple phylogenetically linked *fimH* alleles with silent variations only. The rest are evolutionarily recent FimH variants coded by a single *fimH* allele. In red – the proportion of AIEC strains carrying the corresponding FimH variant. In green – the proportion of non-AIEC strains. (B) Minimum spanning tree based on the MLST allelic profiles portraying the clonal distribution of 45 AIEC strains and 3 reference strains. Each dot represents a given sequence type (ST) and the size of the circle is proportional to the number of strains analyzed. Connecting lines of increasing length and the numbers on these lines demonstrate the number of different alleles between two STs. The color of the dots represents the different AIEC clades and the reference strains.

We used a collection of 92 *E. coli* strains comprising 45 AIEC and 25 non-AIEC strains isolated from the ileal mucosa and stools of CD patients and 22 non-AIEC strains from controls. The *fimH* genes were sequenced from the 92 *E. coli* strains and aligned and analyzed for sequence diversity. For comparison, *fimH* of *E. coli* K-12 was added to the alignment and phylogenetic analysis. Among the 92 *E. coli* isolates, 59 distinct *fimH* DNA allelic variants were identified (**Table S1 in Text S1**). We used Zonal Phylogeny to construct a FimH protein tree from the maximum likelihood DNA tree, collapsing branches with silent changes (**Figure 1A**). This allows identical amino acid changes that are evolutionarily distinct (phylogenetically unlinked) to be readily identified. As a result, the convergent molecular evolution of proteins can be determined directly *via* repeated hotspot mutations in the same amino acid position. Along the protein tree, a total of 42 evolutionarily distinct FimH variants (**Figure 1A**) were identified, and these formed two major clades: the most common, consensus structure (right side of the tree, **Figure 1A**) and a FimH that differed from the consensus sequence by N70S and S78N substitutions (S70/N78 clade, left side of the tree, **Figure 1A**). Seven of the FimH variants were evolutionarily fixed, and the rest are evolutionarily recent. AIEC isolates carried 29 FimH variants (**Table 1**), and non-AIEC isolates carried 22 variants (non-significant difference), with 8 variants carried by both strain types.

**Table 1:** FimH variants of the AIEC isolates studied.

			Mutations at amino acid residue^b^																					
FimH clade	Variants^a^	Strains	Leader peptide		Lectin domain														Pilin domain					
			6	10	10	25	26	27	66	70	73	78	106	109	111	119	129	158	163	166	195	243	261	273
		K12	T	N	V	A	T	V	G	N	G	S	A	L	P	A	L	T	V	R	Y	A	G	G
	**Variant 1**	LF9																						
		LF15																						
		LF25																						
		LF89																						
		LF110																						
	**Variant 2**	LF138						A																
		6281						A																
		6356						A																
	**Variant 3**	6028			A			A																
	**Variant 4**	LF65				T		A																
	**Variant 5**	LF123						A			A													
	**Variant 6**	7093						A			W										F			
	**Variant 7**	LF87						A													F			
	**Variant 8**	LF54						A						W										
**Consensus**	**Variant 9**	LF51						A									R							
	**Variant 10**	7035						A												H				
		7081						A												H				
		7090						A												H				
	**Variant 11**	LF50						A												C				
	**Variant 12**	LF28	N	I				A								V								
		6011	N	I				A								V								
		7022	N	I				A								V								
	**Variant 13**	7074	N	I			S	A								V								
	**Variant 14**	LF26	N					A								V								
		7113	N					A								V								
	**Variant 15**	LF49	N					A								V								A
		LF71	N					A								V								A
		6029	N					A								V								A
		6151	N					A								V								A
	**Variant 16**	6088						A				N								S				
	**Variant 17**	6259						A				N												
	**Variant 18**	LF73						A		S		N												
	**Variant 19**	6254						A			W	N												
	**Variant 20**	LF16						A		S	W	N												
	**Variant 21**	7049						A		S	E	N												
**S70/N78**	**Variant 22**	7136						A		S	R	N												
	**Variant 23**	7103						A		S		N			L									
	**Variant 24**	LF82						A		S		N						P						
	**Variant 25**	7082						A		S		N						A						
	**Variant 26**	6170						A		S		N							A					
	**Variant 27**	6076						A			A	N										V	R	
	**Variant 28**	6283						A	C			N										V		
	**Variant 29**	LF31						A	A	S		N	T											

a Variants are defined as combinations of amino acid mutations.

b Blank entries indicate identity with the wild type. Mutations are indicated.

Ten amino acid positions in FimH were affected by hotspot mutations (**Table 2**). Of the 45 AIEC isolates, 40 carried FimH with a hotspot mutation, while 27 of the 47 non-AIEC isolates were in the same category (P<0.01, **Table 2**). AIEC-specific hotspot mutations were at positions G73, T158 and R166 (P<0.001, **Table 2**). In addition, 33 AIEC isolates carried evolutionarily recent variants, while most non-AIEC variants were of an evolutionarily fixed origin (P<0.05, **Table 2**). Of note, one of the FimH variants of recent origin with a hotspot mutation in an AIEC-specific position (T158P) was carried by the reference AIEC strain LF82 (**Figure 1A**).

**Table 2:** Evolution and hotspot mutations in *fimH* from AIEC and non-AIEC strains.

	AIEC	Non-AIEC
**Evolutionarily fixed FimH variants**	12	29**
**evolutionarily recent FimH variants**	33	17**
**Hotspot mutations in FimH**		
**Total**	**40**	**27** *
T-6N	10	8
A27T/V	7	7
G66A/C/S	1	2
S70N	4	5
**G73A/E/R/W**	**7**	**0** ***
A106T/V	1	1
**T158A/P**	**2**	**0** ***
V163A/I	1	3
**R166C/H/S**	**5**	**0** ***
Y195F	2	1

* **χ^2^ P<0.05.**

** **χ^2^ P<0.01.**

*** **2×2 χ^2^ P<0.001 for 3 hotspot mutations in FimH (specific to AIEC) compared to the rest.**

Thus, in contrast to non-AIEC isolates, AIEC isolates tend to carry FimH with hotspot mutations that are of evolutionarily recent origin and that can be signatures of pathoadaptive mutations.

### MLST analysis and phylotyping

The correlation between *fimH* sequences, multi-locus sequence typing (MLST) and PCR phylogrouping (ABD typing) was established to evaluate the evolutionary relationships among closely related AIEC strains. A total of 24 sequence types (STs) were identified, 3 of which were newly discovered STs (**Table S2 in Text S1**). MLST showed a broad diversity within the various allelic profiles indicating no AIEC-specific genomic background, as shown by MLST ST or ST complex (**Figure 1B**). There were no significant differences between AIEC and non-AIEC isolates in their associations with the major phylogenetic groups, with the B2 group being the largest in both (**Table 3**). All but one FimH variant from the S70/N78 clade was associated with the B2 isolates. B2 strains were also found in the consensus FimH clade (**Table 3**). However, on the basis of MLST allelic profiles, all AIECs from the S70/N78 clade, with very few exceptions, were clearly separated from the AIEC FimH consensus clade (**Figure 1B**).

**Table 3:** Phylotype of *E. coli* according to *fimH* clades.

		Phylotype			
Clade	Number of strain	A	B1	B2	D
**Consensus**	67	10 (28.8%)	7 (10.4%)	28 (41.8%)	13 (19.4%)
**S70/N78**	25	0 (0.0%)	0 (0.0%)	24 (96.0%)	1 (4.0%)

Although the MLST data showed no strong association among the major phylogenetic groups of *E. coli* and AIEC strains, the overall findings demonstrate a clear separation of AIEC within the two FimH major clades, the consensus and the S70/N78.

### Adhesion level of AIEC on intestinal epithelial cells depends on FimH mutations

**Figure 2:**
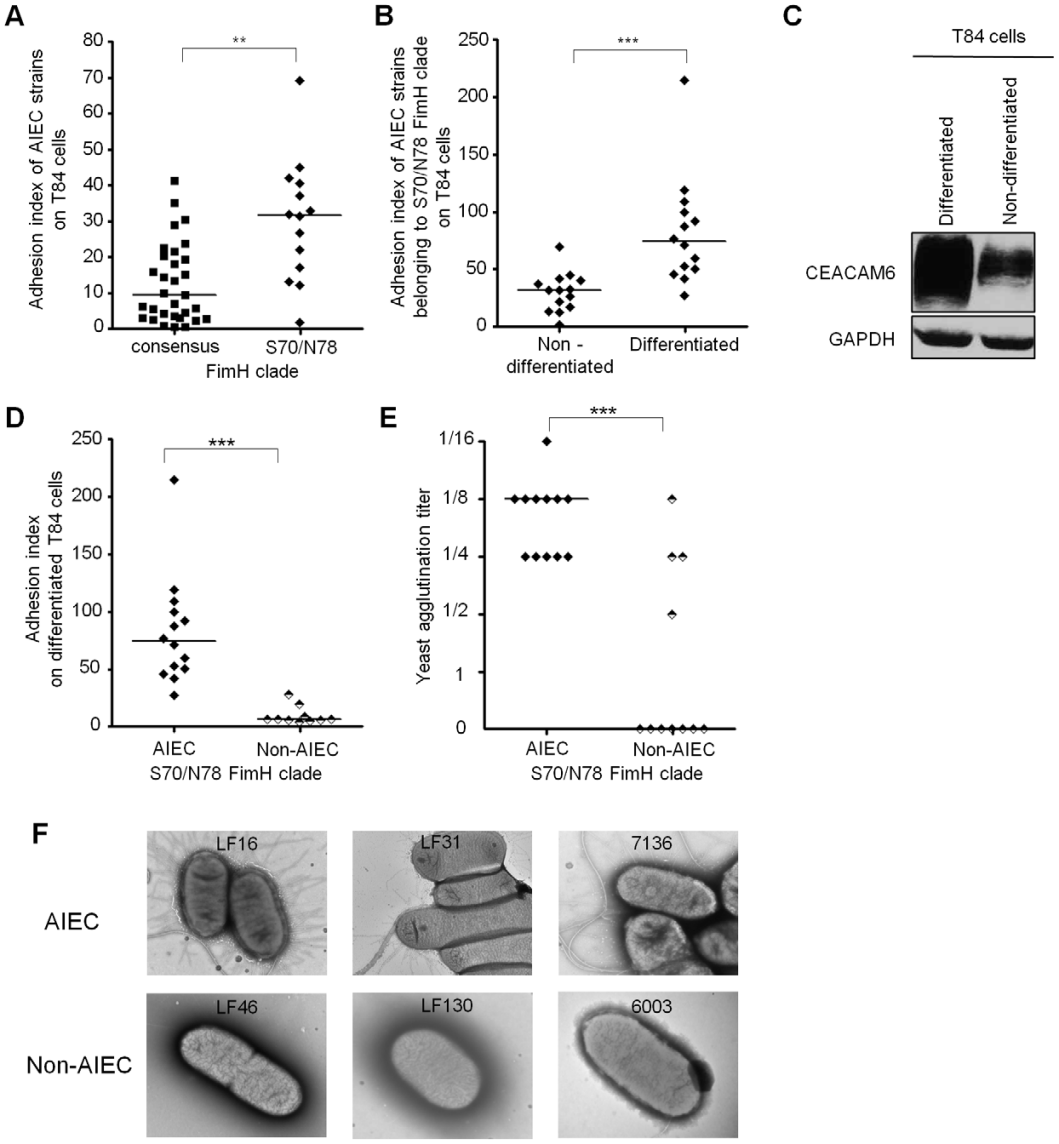
Adhesion ability of AIEC and non-AIEC strains with regard to *fimH* clade and CEACAM6 expression. (A) Cell-associated bacteria were quantified using non differentiated T84 cells after a 3 H infection period, and results were analyzed with regard to *fimH* clade. (B) Cell-associated AIEC bacteria belonging to the *fimH* S70/N78 clade were quantified using undifferentiated and differentiated T84 cells after a 3 H infection period. (C) Western blot analysis of whole protein extracts from non-differentiated and differentiated T84 cells using anti-CEACAM6 and anti-GAPDH antibodies. (D) Cell-associated bacteria (AIEC and non-AIEC bacteria belonging to the *fimH* S70/N78 clade) were quantified using differentiated T84 cells after a 3 H infection period. (E) Yeast agglutination titer of AIEC and non-AIEC strains belonging to the *fimH* S70/N78 clade. (F) Transmission electron micrograph of negatively stained AIEC and non-AIEC bacteria belonging to the *fimH* S70/N78 clade, magnification ×25 000.

FimH adhesin of type 1 pili, which mediates adhesion to cultured intestinal epithelial cells, plays an essential role in the virulence of AIEC strain LF82 [11], [27]. Thus, we investigated the involvement of FimH amino acid substitutions in the adhesion of AIEC and non-AIEC strains to T84 intestinal epithelial cells. Interestingly, a significantly higher ability to adhere to CEACAM6-expressing undifferentiated T84 intestinal epithelial cells was observed for AIEC strains belonging to the S70/N78 clade (including AIEC LF82 reference strain) (**Figure 2A**). When we compared the ability of AIEC strains belonging to the S70/N78 clade to adhere to undifferentiated or differentiated T84 intestinal epithelial cells, we observed a higher adhesion to differentiated T84 cells (**Figure 2B**), which have overexpressed CEACAM6 receptor on the cell surface (**Figure 2C**). Non-AIEC strains from the S70/N78 clade had a low ability to adhere to T84 intestinal epithelial cells compared with AIEC strains from the S70/N78 clade (**Figure 2D**). This was due to the lack of type 1 pili expression as shown by yeast agglutination (**Figure 2E**) and electron microscopy (**Figure 2F**). Of note, the orientation of the *fimS* invertible element, which is phase variable, was mainly in the OFF phase in non-AIEC strains, which would explain the absence of *fim* operon expression (**data not shown**).

These results show that both expression of type 1 pili and FimH amino acid substitutions are required to confer upon AIEC bacteria a greater ability to adhere to intestinal epithelial cells expressing the CEACAM6 receptor.

### Amino acid substitutions affect adhesion to intestinal epithelial cells

**Figure 3:**
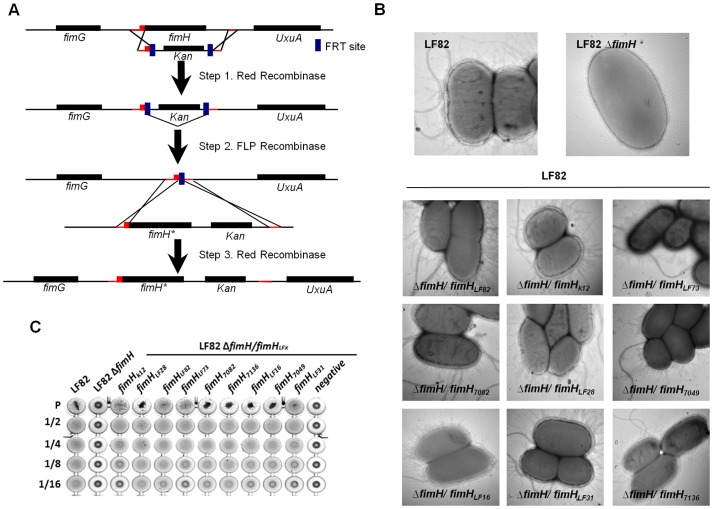
Construction of *fimH* chromosomal mutants. (A) Construction of *fimH* chromosomal mutants. (B) Electron microscopy examination of AIEC LF82 bacteria, LF82-Δ*fimH* isogenic mutant, LF82-Δ*fimH/fimH_LF82_*, LF82-Δ*fimH/fimH_K12_*, LF82-Δ*fimH/fimH_7082_*, LF82-Δ*fimH/fimH_LF28_*, LF82-Δ*fimH/fimH_LF16_*, LF82-Δ*fimH/fimH_7049_*, LF82-Δ*fimH/fimH_LF31_*, LF82-Δ*fimH/fimH_7136;_* LF82-Δ*fimH/fimH_LF73_* showing flagella and type 1 pili expression (magnification ×40 000). (C) Yeast agglutination titer of AIEC LF82 bacteria, LF82-Δ *fimH* isogenic mutant and *fimH* chromosomal mutants.

To determine whether the increased ability of AIEC strains to adhere to intestinal epithelial cells was linked to amino acid substitutions in FimH and was not a consequence of other differences in the bacterial genome, we assessed the effect of FimH variant expression in the genetic background of AIEC LF82 on the ability to adhere to T84 cells. Thus, in the background of *E. coli* LF82, we constructed isogenic strains expressing FimH from LF82 and, as a comparison, FimH from *E. coli* K12, which, unlike the FimH_LF82_, belongs to the consensus FimH clade (with A27V substitution) and to an evolutionarily fixed rather than recent node on the tree (**Figure 3A**). Compared with the wild-type LF82 strain, these constructs had a similar ability to express type 1 pili on the bacterial surface (**Figure 3B**) and to agglutinate yeast (**Figure 3C**), suggesting expression of functional type 1 pili. Other isogenic mutants harboring FimH from other AIEC strains were created in the LF82 background and showed similar patterns of functional type 1 pili expression as shown by electron microscopy examination (**Figure 3B**), yeast agglutination (**Figure 3C**) and colony immunoblotting (**Figure S1**), indicating that all mutations in the *fimH* gene tested in this study do not modify FimH expression.

**Figure 4:**
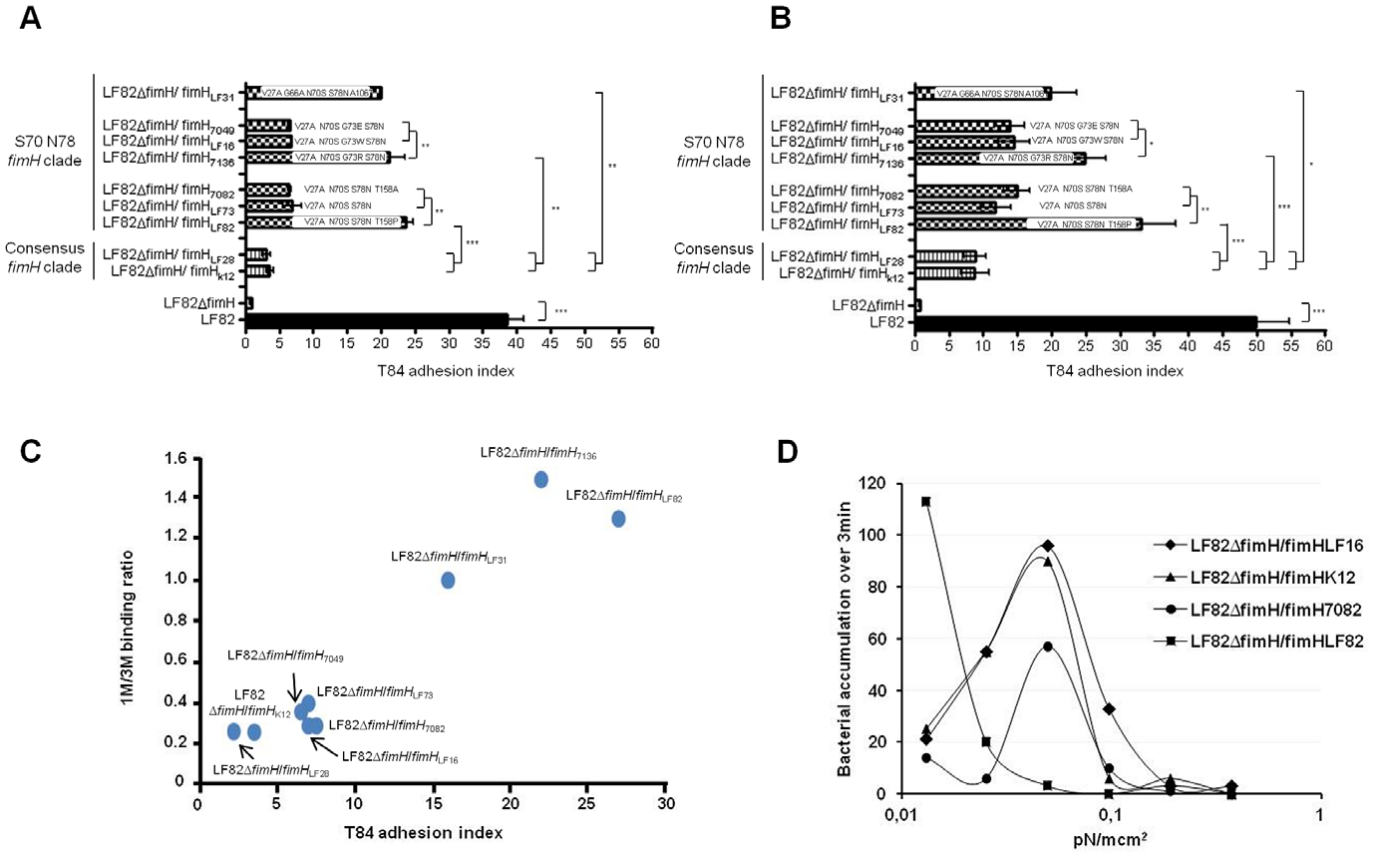
Impact of FimH amino acid substitutions on AIEC adhesion ability. Cell-associated bacteria were quantified using undifferentiated (A) or differentiated (B) T84 cells after a 3 H infection period. WT represents the original AIEC LF82 reference strain and Δ*fimH* represents the LF82-Δ*fimH* isogenic mutant. The other constructs were made in the LF82-Δ*fimH* isogenic mutant, in which various *fimH* variants were reintroduced at the *fimH* locus. Each value is the mean ± SEM of at least four separate experiments (* P<0.05; ** P<0.001; *** P<0.001). (C) Relative ability to bind Man1 (1 M/3 M binding ratio) with regard to undifferentiated T84 index adhesion of the various *fimH* chromosomal mutants constructed. (D) Bacterial binding to 1M-BSA under various shear stresses (pN/m cm^2^) was evaluated by measuring bacterial accumulation over 3 min.

Inactivation of *fimH* in the LF82 strain prevented adhesion to T84 cells (**Figure 4A**). We verified that the replacement of original *fimH* by *fimH*_LF82_ in the AIEC LF82 genome restored most of its ability to adhere to T84 intestinal epithelial cells. In contrast, the replacement of *fimH*_LF82_ by *fimH*_K12_ caused a 6 fold decrease in adhesion to T84 cells compared with the adhesion level mediated by *fimH_LF82_*. *fimH*_LF82_ was replaced by other *fimH*_variants_ in the AIEC LF82 genome to assess their ability to adhere to T84 cells. Constructs expressing an amino acid substitution at AIEC-specific positions (158 and 73) in the background of the S70/N78 variant had a higher adhesion index than the strain harboring *fimH*_K12_. Compared with that of *fimH_L82_*, the effect was variable. For position 158, FimH_LF73_ (with no change in position T158) and FimH_7082_ (with T158A) led to a 2 to 3 fold lower ability to adhere to T84 cells compared with FimH_LF82_ (*i.e.*, with T158P). For position 73, FimH_7136_ (with G73R) had almost the same effect as FimH_LF82_, whereas G73W and G73E substitutions had significantly lower effects on adhesion ability. For position 106, FimH_LF31_ (with A106T combined with G66A) also led to increased ability to adhere to T84 cells at a level similar to that of FimH_LF82_ (**Figure 4A**). As differentiated T84 cells express a higher level of CEACAM6 than undifferentiated T84 cells, we measured the adhesion levels of all the constructs using differentiated T84 cells. For all the constructs tested, the adhesion index was higher for differentiated than for undifferentiated T84 cells (**Figure 4B**). As previously shown, mutations in FimH increase *E. coli* binding to human urinary tract epithelial cells [17], [18], [28]. Examination of the binding of the different FimH constructs to human T24 bladder epithelial cells (**Figure S2-B**) revealed an overall high correlation (R^2^ = 0.69) between the binding to the intestinal and bladder epithelial cells, with the strongest binding exhibited by the FimH_LF82_ variant. However, the relatively strong binding of the 7136 and LF31 variants to the intestinal cells was not as straightforward as with the bladder epithelial cells. This indicates that the specific FimH polymorphisms selected in our AIEC collection, although they have an overall similar effect on the binding to uroepithelial cells, may preferentially enhance adhesion to intestinal epithelial cells.

FimH variants are able to mediate a highly variable ability to bind terminal single mannose residues (monomannose, 1 M) under static adhesion conditions [29]. Low 1M-binding under static conditions corresponds to shear-dependent adhesion of FimH, whereas high 1 M binding is indicative of shear-independent binding properties. We tested the correlation between the ability of FimH variants to bind the intestinal cells and to interact with 1 M. We used 1M-BSA immobilized on the surface of a 96-well plate and compared the level of binding by growth assay. To avoid any potential effects of the differential growth rate of different strains and potential differential fimbrial expression among the FimH variants, the quantitative differences in 1 M binding capabilities of FimH variants were normalized to the oligomannose substrate (3 M binding, as in bovine RNAse B) that is uniformly strong among different FimH variants and directly correlates to fimbrial expression under static conditions. The bacterial ability to bind to T84 cells directly and strongly correlated with FimH-mediated ability to interact with 1 M (R^2^ = 0.92) (**Figure 4C**). FimH variants with low 1 M binding mediated low binding to T84 cells, whereas FimH variants with high binding to mannose mediated correspondingly higher cell binding. We tested the binding of a subset of FimH variants to 1 M-BSA and found that the FimH_LF82_ that exhibited the strongest 1 M binding under static conditions completely lost dependency on shear (**Figure 4D**), with the strongest binding mediated under the lowest shear that diminished rapidly with shear increase. In fact, the ability of the FimH_LF82_ variant to bind mannose under static conditions is comparable to the strongest 1M-binding phenotype observed previously with different naturally occurring FimH variants [30]. In contrast, the FimH_7082_ and FimH_LF16_ variants that demonstrated low 1 M binding under static conditions showed strong shear-dependence of the binding, similar to that of the FimH_K12_.

Thus, the differential adhesion to intestinal epithelial cells by *E. coli* was due to polymorphisms in FimH, with the adhesin variant from AIEC LF82 showing the highest binding effect. Additionally, binding of FimH was correlated with the ability to bind to the terminal single mannose residues present in the N-linked oligosaccharide moieties of the cell surface CEACAM glycoproteins.

### Polymorphisms in FimH affect intestinal colonization, extraintestinal dissemination and ability to trigger inflammation

**Figure 5:**
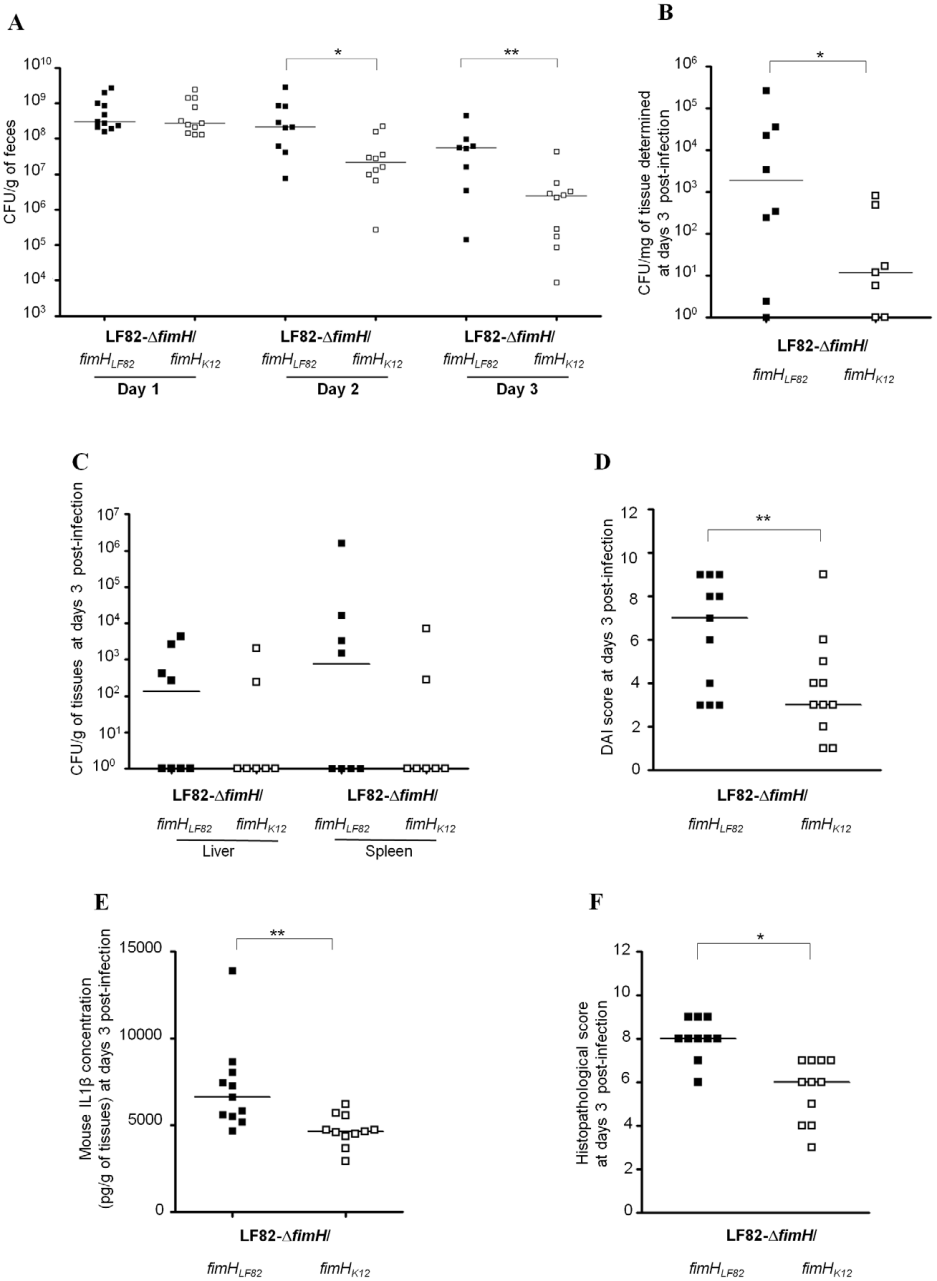
Bacterial colonization, colonic mucosa inflammation and translocation in CEABAC10 mice infected with LF82*ΔfimH/fimH_LF82_* or LF82*ΔfimH/fimH_K12_* mutants. (A) Quantification of LF82-Δ*fimH/fimH_LF82_* (black square) or LF82-Δ*fimH/fimH_K12_* (white square) bacteria in the feces of CEABAC10 mice receiving 0.25% DSS in drinking water after oral infection with 10^9^ bacteria on day 0. (B) Quantification of colonic mucosal-associated LF82-Δ*fimH/fimH_LF82_* or LF82-Δ*fimH/fimH_K12_* bacteria on day of sacrifice. (C) Quantification of bacteria on day of sacrifice in the liver and spleen. (D) DAI was performed for CEABAC10 transgenic mice infected with (black square) LF82*ΔfimH/fimH_LF82_* or with (white square) LF82*ΔfimH/fimH_K12_*. (E) Il-1β secretion by colonic mucosa. (F) Histopathological scoring for several parameters of colonic inflammation was performed for CEABAC10 transgenic mice infected with (black square) LF82*ΔfimH/fimH_LF82_* or with (white square) LF82*ΔfimH/fimH_K12_*. * P<0.05; ** P<0.01; and *** P<0.001.

To investigate the role of FimH polymorphisms in gut colonization by AIEC LF82, CEABAC10 transgenic mice expressing human CEACAM molecules (CEACAM3, CEACAM5, CEACAM6 and CEACAM7) were challenged with AIEC LF82-*ΔfimH* expressing either *fimH* belonging to the N70/S78 *fimH* clade (*fimH_LF82_* and fimH7082) or *fimH* belonging to the consensus clade (*fimH_K12_ and fimHLF28)*. Quantification of AIEC LF82 bacteria in stool samples on post-infection day 2 revealed a 21.5 fold decrease (P = 0.01) in LF82-*ΔfimH*/*fimH_K12_* (2.6×10^7^ CFU/g of feces) compared with LF82-*ΔfimH*/*fimH_LF82_* (5.5×10^8^ CFU/g of feces, **Figure 5A**) and a 20.6 fold decrease at day 3 post-infection (2.9×10^6^ vs 6.0×10^7^ CFU/g of feces, respectively; P = 0.008). A decreased ability to adhere to the colonic mucosa of transgenic mice was observed for LF82-Δ*fimH/fimH_K12_* (1.2×10^1^ CFU/g of tissues, **Figure 5B**) compared with LF82-Δ*fimH/fimH_LF82_* (3.5×10^2^ CFU/g of tissues) at day 3 post-infection. In addition, dissemination of LF82Δ*fimH/fimH_LF82_* was observed in the liver and/or the spleen of four transgenic mice (**Figure 5C**), whereas LF82-Δ*fimH/fimH_K12_* was found to disseminate to the liver and the spleen of only two transgenic mice. These results suggest that the replacement of *fimH_LF82_* by *fimH_K12_* in the AIEC LF82 genome decreased the ability of bacteria to persist in the gut of CEABAC10 mice.

The disease activity index (DAI) score and histological analyses of colonic tissue were performed at day 3 post-infection to assess the degree of inflammation. A significantly decreased DAI score (P = 0.042) was observed for mice infected with LF82-Δ*fimH/fimH_K12_* (4.0±2.6), LF82-Δ*fimH/fimH_7082_* and LF82-Δ*fimH/fimH_LF28_* compared with mice infected with LF82-Δ*fimH/fimH_LF82_* (7.0±3.4) (**Figure 5D**). The presence of blood in stools was only observed in transgenic mice infected with LF82-Δ*fimH/fimH_L82_*. Of note, type 1 pili are not the only virulence factor of AIEC LF82 because mice infected with LF82-Δ*fimH/fimH_K12_* presented with mild gut inflammation. Cytokines were also quantified in colonic specimens after sacrifice at day 3 post-infection. Levels of the pro-inflammatory cytokine IL-1ß were significantly higher in mice infected with LF82-Δ*fimH/fimH_LF82_* than in mice infected with LF82-Δ*fimH/fimH_K12_* (P = 0.025; **Figure 5E**). However, no significant difference was observed for IL-6 and KC (Keratinocyte-derived Cytokine) (**data not shown**). In addition, examination of colonic mucosa revealed that transgenic mice infected with LF82-Δ*fimH/fimH_K12_* had lower infiltration of inflammatory cells and less severe epithelial damage with less surface affected than mice infected with LF82-Δ*fimH/fimH_LF82_*. The colonic histological score was significantly lower for mice infected with LF82-Δ*fimH/fimH_K12_* (6.0±1.4) than for mice infected with LF82-Δ*fimH/fimH_LF82_* (8.0±0.9) (P = 0.008; **Figure 5F**).

**Figure 6:**
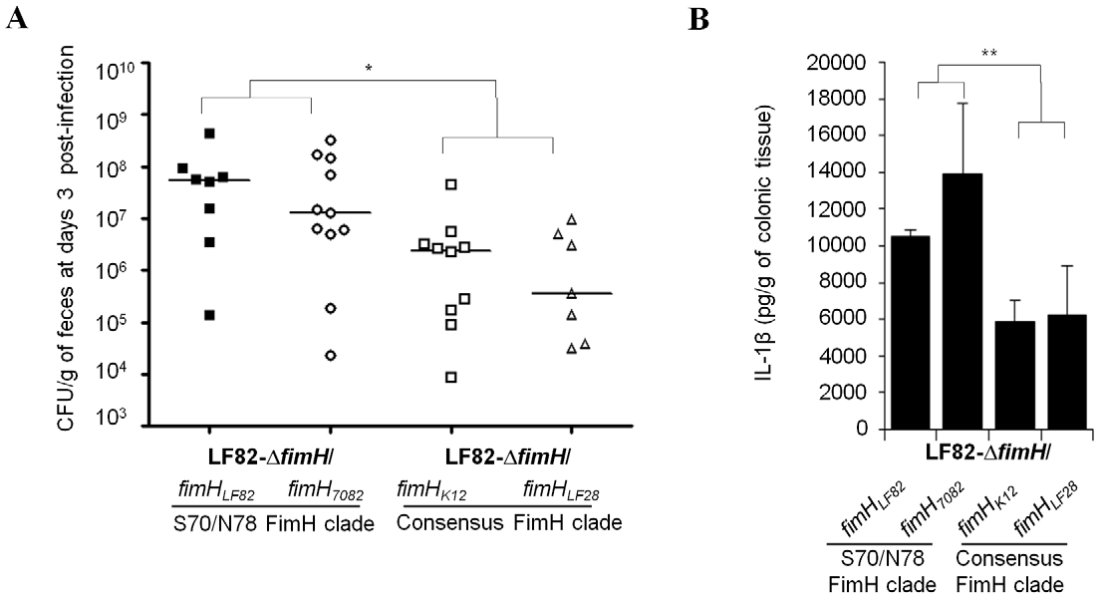
Bacterial persistence and IL-1β secretion by colonic mucosa of CEABAC10 mice according to FimH sequence. (A) Quantification of LF82-Δ*fimH/fimH_LF82_* (black square), LF82-Δ*fimH/fimH_7082_* (white circle), LF82-Δ*fimH/fimH_K12_* (white square) and LF82-Δ*fimH/fimH_LF28_* (white triangle) bacteria in the feces of CEABAC10 mice infected with 10^9^ bacteria on day 3 post-infection. (B) Quantification of IL-1β release by colonic loops infected with LF82 bacteria expressing *fimH* from LF82 or 7082 (S70/N78 clade) and from K12 or LF28 (consensus clade). * P<0.05; ** P<0.01.

Quantification of AIEC LF82 bacteria in stool samples on day 3 post-infection revealed no differences between LF82 expressing *fimH* from LF82 or 7082 and between LF82 expressing *fimH* from K12 or LF28. However, a significant decrease (P = 0.01) in LF82-*ΔfimH*/expressing *fimH* consensus cluster compared with LF82-*ΔfimH*/expressing N70/S78 clade was observed (**Figure 6A**). Of interest, the levels of the pro-inflammatory cytokine IL-1ß secreted by colonic tissues were significantly higher when colonic loops were inoculated with LF82 bacteria expressing *fimH* from LF82 or from 7082 compared with LF82 bacteria expressing *fimH* from K12 or from LF28 (**Figure 6B**).

Altogether, these results show a major role for polymorphisms in FimH adhesin of AIEC in the GI tract *in vivo* and demonstrate that such FimH polymorphisms contribute to colitis and gut inflammation in CEABAC10 mice.

## Discussion

Abnormal expression of CEACAM6 is observed at the apical surface of the ileal epithelium in CD patients, and CD ileal lesions are colonized by pathogenic adherent-invasive *Escherichia coli* (AIEC) [3], [11]. CD-associated AIEC colonize and induce strong gut inflammation in transgenic mice expressing human CEACAMs, which act as a receptor for type 1 pili produced by AIEC bacteria [11], [31]. AIEC also induce CEACAM6 expression by intestinal epithelial cells directly by adhering to host cells and indirectly *via* increased secretion of TNF-α from AIEC-infected macrophages. Our hypothesis was that abnormal expression of CEACAM6 in the ileal mucosa of CD patients can select for *E. coli* strains harboring pathoadaptive mutations in FimH adhesin that enhance binding to mannosylated receptors. Knowledge of *fimH* polymorphisms is critical for our understanding of the mechanisms of AIEC gut colonization in CD patients and for efforts to develop novel therapeutic strategies. Indeed, as observed with uropathogenic *E. coli* (UPEC) in the context of urinary infections [32], blocking the interaction between type 1 pili and CEACAM molecules might serve as a specific means of disrupting colonization and the subsequent inflammatory amplification loop.

The phylogeny of 92 *E. coli* isolates (45 AIEC and 47 non-AIEC) from a French collection was investigated using a combination of PCR ABD typing, MLST and DNA sequencing for *fimH* SNPs analysis. The number of FimH amino acid variants was significantly higher in AIEC strains than in non-AIEC strains. In addition, most of the AIEC strains with similar amino acid variants belonged to the same phylogroup. Similar observations were recently reported in UPEC [33]. Of interest, AIEC LF82 genome sequencing has revealed that this strain is genetically similar to UPEC strains [34]. MLST analysis of seven housekeeping genes identified 24 different STs among the 45 AIEC strains analyzed. Some of these clustered together with the urinary tract pathogen (CFT073 and UTI89) and the avian pathogen (APEC O1:K1/H7). This is in agreement with a study based on MLST analysis reporting that AIEC from a Canadian cohort did not evolve from a single ancestral background [35]. In general, *E. coli* from A and B1 groups are less pathogenic, whereas B2 and D strains are more frequently pathogenic, causing urinary tract and other extraintestinal infections [36], [37]. On the basis of *fimH* gene sequences, AIEC can be divided into two clades, composed of the primary consensus or of S70/N78 FimH variants. This separation into two clades was confirmed by our MLST data. Most of the AIEC strains tested in the present study harboring a FimH of the S70/N78 clade belonged to the B2 phylogroup, which is consistent with findings of a previous study that reported that the N70S/S78N amino acid combination in FimH is associated with the B2 phylogroup [19]. MLST analysis and phylogrouping (ABD typing) of the AIEC collection used in this study are in accordance with those of previous studies [37], [38] and support the observation that AIEC isolated from CD patients tend to belong to B2 or D phylotypes. This suggests that these isolates have taken advantage of a specific micro-environment found in IBD gut.

In addition to detecting hotspot mutations, zonal phylogeny analysis can determine the relative evolutionary timing of the emergence of protein variants by distinguishing evolutionarily fixed or ‘old’ FimH variants (encoded by alleles with silent diversity) and evolutionarily recent variants (coded by just a single allele, without silent diversification). Pathoadaptive changes in bacterial proteins are specifically associated with hotspot mutations of recent origin due to the source-sink dynamics of virulence evolution. Thus, in AIEC bacteria, *fimH* pathoadaptive mutations may have arisen several times during evolution. This is in accordance with results reported by different groups showing that FimH adhesin has accumulated recent amino acid replacements, which increase tropism for the uroepithelium [18], [28], [30], [39]. While FimH variations could potentially affect fimbrial biogenesis [40], the differential binding to mannose observed in our study cannot be explained by FimH expression levels. First, it has been shown previously that natural mutation-induced 1M-specific binding is independent of FimH surface expression [30]. Secondly, we did not observe a significant difference in the piliation level of bacteria expressing different FimH variants. Finally, in this study, the variable 1M-binding was normalized to 3M-binding that is strong for different FimH variants and directly reflects the piliation level [18]. Most of these amino acid replacements increase the monomannose binding capability of FimH under low shear by altering the allosteric catch bond properties of the protein [41].

**Figure 7:**
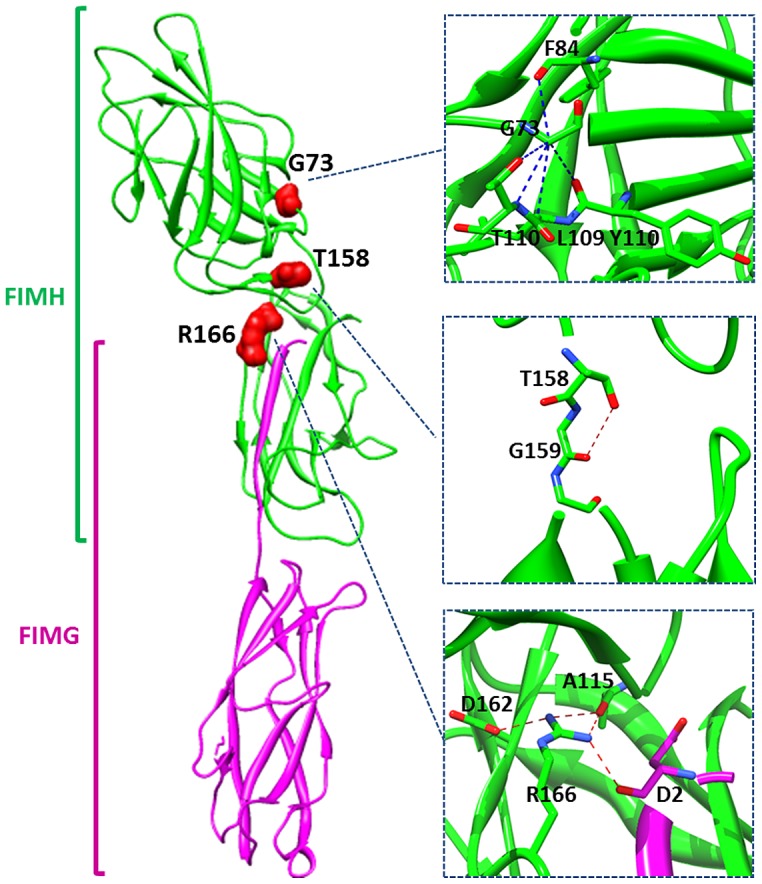
Positions 73, 158 and 166 in the crystal structure of FimH in complex with FimG (pdb id: 3JWN). Carbon atoms are shown in green for FimH and in violet for FimG. Oxygen atoms are shown in red and nitrogen atoms in blue. The hydrogen bonds are shown in red dashed lines, and close contact in blue dashed lines.

FimH is the tip adhesin of mannose-specific type 1 pili, which are required for AIEC to colonize the gut mucosa of transgenic CEABAC10 mice expressing human CEACAMs [31]. FimH mediates binding to mannosylated CEACAM receptors present on the colonic mucosa luminal surfaces of CEABAC10 mice and human intestinal epithelial cells. This binding is thought to be the primary molecular feature by which FimH promotes colitis [11]. In the 45 AIEC strains used in this study, we identified 22 amino acid substitutions in FimH that were distributed throughout the protein structure. Eight amino acids of FimH form a binding pocket that interacts with D-mannose by hydrogen bonding and hydrophobic interactions [42]. In all 92 strains tested (AIEC and non-AIEC), the mannose binding pocket was conserved. Similar observations were reported in a UPEC strain collection [43], which indicates that the mannose binding pocket of FimH adhesin is perfectly designed to engage with a monosaccharide receptor and that amino acid substitutions in this pocket are not selected in pathogenic *E. coli* because they are likely to disrupt, rather than enhance, binding to mannose. However, we found amino acid substitutions in AIEC strains belonging to the S70/N78 clade that were located near the binding pocket and the interdomain linker between the pilin and lectin domains. Interestingly, AIEC belonging to the S70/N78 clade showed a higher ability to adhere to undifferentiated or differentiated T84 intestinal epithelial cells. This was observed after both short (as shown in **Figure S2-A**) and long periods of bacteria-cell interaction, indicating that the potential signaling and regeneration initiated during the time of exposure to bacteria does not modify the differences between all the mutants, as previously described [44]. In addition, a high but not complete correlation between the binding of the FimH constructs to intestinal and bladder epithelial cells was observed. This suggests that FimH polymorphisms might be partly associated with intestinal binding, but it is also possible that FimH polymorphisms may be involved in increased fitness in intestinal colonization and in bladder binding. Among the hotspots of amino acid substitutions found in the S70/N78 clade, positions G73, T158 and R166 are crucial for increasing bacterial adhesion to T84 cells (**Figure 7**). Substitutions at these positions can induce subtle modifications in FimH organization and thereby modify its functionality. The Cα atom of the G73 residue, which harbors no side chain, is located in close contact to residues 108 to 110. Its substitution by glutamic acid can lead to steric constraints that modify the conformation of FimH locally. Substitution of T158P replaces a residue donor and/or acceptor of hydrogen bonds with a proline, a residue harboring a five-membered ring, which imposes rigid constraints. The side chain of R166 is involved in a hydrogen bond network that links the pilin domain (R166) of FimH to its lectin (A115) and pilin (D162) domains as well as FimG (D2). The substitution of R166 breaks this network and may therefore affect the interactions between these domains [45], [46]. All these modifications are located in the vicinity of the interdomain zone, which plays an important role in the catch bond mechanism of FimH adhesion [47]. FimH exists in two distinct functional states: one with relatively weak mannose binding and another with strong mannose binding [16], [48]. It was predicted that the catch bond mechanism of shear-enhanced bacterial adhesion involves conversion from the weak state into the strong state via extension of the interdomain linker chain by drag-originated tensile force and that the linker chain extension is allosterically linked to the strong binding site conformation [16], [48]. The 3 substitutions may therefore affect these dynamic processes and be the result of the same process, the aim of which is the pathoadaptation of the FimH interdomain zone, which improves mannose binding in the CD ileal tract. We therefore investigated the functional effect of FimH mutations under various shear stresses. The amino acid substitutions G73R, A106T and T158P led to very high binding, which is shear-independent. In contrast, T158A (FimH_7082_) and G73W (FimH_LF16_) amino acid substitutions led to shear-dependent low binding. A recent study argued that the V27A substitution (*i.e.*, with alanine in position 27) is the primary pathoadaptive FimH mutation arising in AIEC isolated from CD pediatric patients in the entire spectrum of mucosal inflammation [49]. However, our data show that A27 is found in most natural FimH variants, including the consensus sequence, S70/N78 primary variants and FimH from non-AIEC. Thus, V27A is unlikely to be pathoadaptive for AIEC in CD, as it is not sufficient to mediate a high level of bacterial binding to CEACAM receptors. In contrast, additional mutations, such as T158P (in the A27 background), which is not found in AIEC isolated from pediatric patients [49], enable FimH to strongly bind to T84 intestinal epithelial cells and could confer advantage in the context of an inflamed mucosa. This also suggests that shear might not play a significant role in the binding of *E. coli* to intestinal lesion sites, where conditions instead select for strong shear-independent binding.

In a mouse model, it was observed that the advantage of mutator bacteria when colonizing a new host is due to their ability to generate adaptive mutations rapidly, enabling them to exploit the ecosystem resources more quickly than wild-type bacteria [50]. For example, some natural bacterial isolates, such as those of *Pseudomonas aeruginosa* found in the lungs of cystic fibrosis patients, have a strong mutator phenotype. In the case of IBD patients, owing to prolonged disease duration, it is possible that AIEC evolves and adapts to colonize gut mucosa by FimH mutability. Phylogenetic analysis of *fimH* sequences delineated a tight S70/N78 clade containing LF82, the reference strain for AIEC. Interestingly, UPEC (strain CFT073) and avian pathogenic *E. coli* (strain O1:K1:H7) were also in this S70/N78 clade. This raises the possibility that IBD-isolated *E. coli* are members of a general pool of extraintestinal pathogenic *E. coli* that reside in the gut and have evolved specific potentialities dependent upon their microenvironment. Allelic replacement of the *fimH* gene in the AIEC LF82 genetic background by *fimH* from strains belonging to the FimH consensus clade (non-pathogenic *E. coli* K12 and LF28) significantly decreased the ability of bacteria to colonize the gut mucosa of transgenic CEABAC10 mice expressing human CEACAM6 and consequently decreased the ability of AIEC bacteria to trigger colitis (weight loss, diarrhea, presence of blood in stools), histological damage to intestinal mucosa and pro-inflammatory interleukin 1-beta secretion by the colonic loop. Similarly, it has been reported that mutated FimH variants conferred an advantage upon UPEC isolates in colonization of the urinary tract in a mouse model and correlated with the overall extraintestinal virulence of *E. coli* [19], [28]. FimH is under positive selection in clinical isolates of UPEC, which is consistent with its critical role in human urinary tract infection [43], [51]. Because antibiotic resistance is rising among uropathogens, the identification of FimH as a critical factor during UPEC infection provides avenues for the development of novel preventative measures against these infections, such as vaccines targeting FimH, the development of mannoside compounds, or, more recently, the design of biarylmannose-derivative FimH antagonists (for review, [32]). Based on our results, we could develop similar therapeutic strategies for preventing AIEC colonization in CD patients.

This study reinforces a long-standing hypothesis that the dynamics of pathogen genomes are important for infectious disease processes. In general, data from comparative genomics support the hypothesis of widespread involvement of horizontal gene transfer in the evolution of *E. coli*, leading to the presence of distinct and variable ‘genomic islands’ within the conserved ‘chromosomal backbone’ in several bacterial lineages. In this study, we demonstrate that not only mobile genetic modules but also point mutations facilitate the rapid adaptation of *E. coli*, particularly AIEC strains, to changing environmental conditions and hence extend the spectrum of infection sites. These specific allelic variants in the *fimH* gene could be a hint of specific *E. coli* adaptation to the inflammatory state and could represent promising new targets for molecular characterization of AIEC. The combination of PCR ABD typing, MLST and *fimH* SNP analysis may be a useful method to predict the potential virulence of *E. coli* isolated from IBD patients for epidemiological studies and to identify new approaches for therapeutic intervention to block interaction between AIEC and gut mucosa in the early stages of IBD.

## Materials and Methods

### *E. coli* collection and phylotyping

The *Escherichia coli* strain collection was isolated from Crohn's disease (CD) patients or non-inflammatory bowel disease (IBD) patients. The *E. coli* strains which had the ability to invade intestine-407 cells and to replicate within macrophages were considered adherent and invasive *E. coli* (AIEC). Forty-five AIEC strains were obtained from CD patients (25 isolated from stools of CD patients and 20 isolated from ileal mucosa), and 47 non-AIEC strains were also obtained (25 isolated from ileal mucosa of CD patients, 7 from ileal mucosa of non-IBD patients and 15 from stools of non-IBD patients). *E. coli* isolates were phylotyped into A, B1, B2 and D groups using a modified triplex polymerase chain reaction (PCR) method [52]. The primers used are shown in **Table 4**. For comparison, sequences of an *E. coli* K12 strain (MG1655, GenBank accession no. NC_000913) and of strains CFT073 (UPEC, NC_004431) and APEC O1 (APEC, NC_008563) were used.

**Table 4:** Primers used in this study.

Target gene	Primer used	Primer sequence (5′ to 3′)	PCR product (bp)	Annealing Temp (°C)	Reference
*E. coli* ABD typing					
*chuA*	chuA.1	GACGAACCAACGGTCAGGAT	279	55	[52]
	chuA.2	TGCCGCCAGTACCAAAGACA			
*tspE*	tspE4C2.1	GAGTAATGTCGGGGCATTCA	211	55	[52]
	tspE4C2.2	CGCGCCAACAAAGTATTACG			
*yjaA*	yjaA.1	TGAAGTGTCAGGAGACGCTG	152	55	[52]
	yjaA.2	ATGGAGAATGCGTTCCTCAAC			
Chromosomal mutant *fimH* strains					
Isogenic mutant *fimH*	MIfimHF	CCCGAAGAGATGATTGTAATGAAACGAGTTATTACCCTGTTTGCTGTACTGCGTAGGCTGGAGCTGCTTCG	1500	55	In this study
	MIfimHR	GCACCTGAGCCTGCCATTGGCAGGCTCTGTTCGGATTGTCGGTAAAGTGCGCCATATGAATATCCTCCTTAG			
fimH amplification	fimHF	CCCGAAGAGATGATTGTAATGAAACGAGTTATTACCCTGTTTGCTGTACTGC	1000	55	In this study
With kanamycin-left	fimHR_km-left	CGAAGCAGCTCCAGCCTACGCCTACAAAGGGCTAACGTGC			
Kanamycin amplification	kmF_fimH-right	GCACGTTAGCCCTTTGTAGGCGTAGGCTGGAGCTGCTTCG	1500	55	In this study
	fimHR_km-left	CGAAGCAGCTCCAGCCTACGCCTACAAAGGGCTAACGTGC			

### Multilocus sequence typing (MLST) and *fimH* sequencing

Multilocus sequence typing (MLST) was performed as previously described by Wirth et al. [37]. Alleles and sequence types (ST) were assigned in accordance with the *E. coli* MLST website (http://mlst.ucc.ie/mlst/dbs/Ecoli). *fimH* was amplified by PCR and Sanger sequenced using the primers listed in **Table 4**.

### Adhesion assay of human intestinal and bladder epithelial cells

T84 cells (derived from human colorectal carcinoma) and T24 cells (human bladder epithelial cells) were purchased from ATCC and maintained in an atmosphere containing 5% CO_2_ at 37°C in appropriate medium. T84 cells were cultured in DMEM/Ham's F12 medium (PAA) supplemented with 10% (vol/vol) fetal calf serum (Lonza, Walkersville, MD USA), 1% L-glutamine (Life-Technologies), 200 U penicillin, 50 mg streptomycin, 0.25 mg amphoterocin B per liter, and 1% hepes buffered saline solution (Lonza), and T24 cells were cultured in McCoy media. Briefly, T84 intestinal epithelial cells were seeded at a density of 2×10^5^ cells/cm^2^ in culture plates (Falcon) for 48 H (undifferentiated) or 21 days (differentiated). Cells were infected during a short (30 minutes) or a long (3 hours) period at a multiplicity of infection of 10 bacteria per cell for adhesion. Infected cells were centrifuged at 900 g for 10 min at 25°C and maintained at 37°C. Cells were washed three times in phosphate-buffered saline (PBS; pH 7.2). The epithelial cells were then lysed with 1% Triton X-100 (Sigma) in deionized water. Samples were diluted and plated onto Luria-Bertani (LB) agar plates to determine the number of CFU corresponding to the total number of cell-associated bacteria. T24 human bladder epithelial cells were grown for 48 H in culture plates to reach confluency. Cells were infected with 2.5×10^6^ bacteria per well and kept at 37°C for 30 min. Cells were washed and lysed as described above. Serial dilutions of the bacteria were plated on LB agar plates for counting. The adhesion index was expressed as the mean number of associated bacteria per epithelial cell.

### Construction of chromosomal mutant *fimH* strains

**Figure 4A** shows construction of *fimH* chromosomal mutants obtained using the red recombinase system described by Datsenko *et al.* [53] and Chaveroche *et al.* [54]: the chromosomal *fimH* gene in LF82 was replaced by a kanamycin (Km) resistance cassette from the pkD4 plasmid [53] using a linear PCR fragment made with primers MIfimHF and MIfimHR (**Table 4**), yielding strain LF82*ΔfimH* (Km^R^). The kanamycin resistance cassette was removed by transient expression of the Flp recombinase from the plasmid pCP20 [53], yielding the strain LF82*ΔfimH* (Km^S^). The pKOBEG plasmid (Chloramphenicol^R^) was transformed into LF82*ΔfimH* (Km^S^), yielding the strain LF82*ΔfimH* (Km^S^, Cm^R^). The *fimH_variant_* gene was amplified from AIEC strains using Platinum Taq high fidelity DNA polymerase (Invitrogen) with primers FimHF and FimHR_Km-left. The kanamycin resistance cassette was amplified from the pKD4 plasmid with primers KmF_fimh-right and MifimHR (**Table 4**). After PCR clean-up (Macherey-Nagel), the two PCR fragments were pooled and used for PCR amplification (Taq high fidelity polymerase, Invitrogen) without primers, yielding a 2.5 kb PCR fragment. The red recombinase system was used to transform the LF82*ΔfimH* (Km^S^ Cm^R^) strain with this 2.5 kb fragment, yielding strain LF82 *ΔfimH/fimH*_variant_ (Km^R^,Cm^S^). Chromosomal integration junctions and the entire *fimH* gene sequence were verified by sequencing for all *fimH* mutant strains.

CEACAM6 expression in T84 cells was measured by immunoblotting experiments as described in Barnich *et al*. [11].

### Yeast agglutination titers

Commercial baker's yeast (*Saccharomyces cerevisiae*) was suspended in PBS (20 mg/mL). Bacterial strains were grown overnight at 37°C without agitation on LB broth, washed, and resuspended in PBS at an optical density of 0.5 at 620 nm. The bacterial suspension was diluted and 50 µL were deposited on a 96-well microplate (Greiner). Equal volumes of yeast cell suspension were added to each well. Aggregation was monitored visually, and the titer was recorded as the last dilution of bacteria giving a positive aggregation reaction.

### Colony immunoblotting

Bacteria grown overnight in LB were harvested, resuspended in PBS at OD_620_ 0.1, and spotted onto nitrocellulose membranes. Membranes were dried and blocked with 5% (w:v) milk in Tris-buffered saline-Tween 0.05% (TBST) at room temperature for 2 h. Membranes were reacted with type 1 pili antiserum diluted 1/1000 in TBST with 2% (w/v) milk overnight at 4°C. Immunoreactants were then detected using a secondary anti-rabbit antibody conjugated with alkaline phosphatase and ECL (Thermo).

### Transmission electron microscopy

Bacteria were grown overnight at 37°C without shaking on LB broth, placed for 1 min on carbon-Formvar copper grids (Electron Microscopy Sciences, Hatfield, England) and negatively stained for 1 min with phosphotungstic acid pH 6.0. Grids were examined with a Hitachi H-7650 transmission electron microscope.

### Bacterial binding

Static assays of bacterial adhesion to immobilized 1 M ligands (Man1-BSA) were carried out in 96-well plates as described previously [29]. To avoid any potential effects of the possibly differential growth rate of different strains, the 1M-binding was normalized to binding to trimannose (oligomannose substrate, as in bovine RNAse B) to which all the FimH variants bind strongly under static conditions. Briefly, the plates were coated with mannosylated substrates in 0.02 M NaHCO3 buffer and blocked with 0.1% BSA in PBS. 100 µl of *E. coli* suspension at A_540_ 2.0 (radiolabeled by growing overnight in the presence of [3H]thymidine) were then incubated in the wells for 45 min at 37°C. After washing away unbound bacteria, the level of bacterial binding was measured by counting the radioactivity in each well. The actual number of bound bacteria was determined from calibration curves. Each data point was done in quadruplicate and then averaged.

Bacterial binding to 1M-BSA was performed as previously described [55]. Surface accumulation of bound bacteria was measured in a field of view (appx. 150×150 micrometers), after 3 min of flow at each shear.

### Infection of mice

Twelve-week-old FVB/N CEABAC10 transgenic male mice (body weight, ≈26–28 g) were pretreated by oral administration of the broad-spectrum antibiotic streptomycin (20 mg intragastric per mouse) to disrupt normal resident bacterial flora in the intestinal tract [56] and were orally challenged with 10^9^ bacteria 24 h later. Animals received a very low dose of 0.25% (wt/vol) of dextran sulfate sodium (DSS; molecular mass = 36,000–50,000 daltons; MP Biomedicals) in drinking water starting 3 days before infection to increase the accessibility of bacteria to the surface of the epithelial layer. When mice attained 80% of their initial weight or 3 days after oral bacterial infection, they were anesthetized with isoflurane and then euthanized by cervical dislocation. Colonic specimens were collected to quantify mucosal-associated bacteria and to analyze histological damage. The spleen and liver were removed to quantify translocated bacteria.

Bacteria interactions were also studied using mouse colonic loops as previously described [57]. Mice were starved for 12 H before surgery, with water available ad libitum. They were anesthetized and their intestines exteriorized through a midline incision. Two colonic segments (approximately 1 cm) were ligated and inoculated by approximately 5.10^7^ bacteria. After a 4 hour period, mice were anesthetized with isoflurane and then euthanized by cervical dislocation. Colonic loops were incubated in DMEM/Ham's F12 medium supplemented with antibiotics (see paragraph on culture cells) for 24 H in an atmosphere containing 5% CO2 at 37°C to measure cytokine release.

One, 2, and 3 days after bacterial infection, fresh fecal pellets (100–200 mg) were collected from individual mice and resuspended in PBS. After serial dilution, bacteria were enumerated by plating on LB agar medium containing 50 mg/µL ampicillin and 20 mg/µL erythromycin to isolate the chromosomal *fimH* mutant of AIEC LF82 and incubated at 37°C overnight. Mucosa-associated AIEC bacteria at day 3 after infection were counted by homogenization of 2 cm of colon and by plating onto LB agar containing appropriate antibiotics and incubated overnight at 37°C.

Colonic damage was ascertained by DAI as defined in Carvalho *et al.* [31] (**Table S3 in Text S1**). Rectal bleeding was assessed by Hemoccult II test (SKD SARL), in which the scores range from 0 (healthy) to 12 (greatest colitis activity). For histological analysis, the entire colon was excised and rolls of the proximal colon were fixed in buffered 4% formalin, paraffin embedded, cut into 5 µm slices, and stained with hematoxylin/eosin/safranin. The histological severity of colitis was graded in a blind fashion by a GI pathologist. The tissue samples were assessed for the extent and depth of inflammation and the extent of crypt damage, as presented in **Table 5**. The histology score corresponds to the sum of all items.

**Table 5:** Histological grading of intestinal inflammation**.**

Symptoms/score	Characteristics
**Infiltration of inflammatory cells**	
0	Rare inflammatory cells in the lamina propria
1	Increased numbers of inflammatory cells, including neutrophils in the lamina propria
2	Confluence of inflammatory cells extending into the submucosa
3	Transmural extension of the inflammatory cell infiltrate
**Infiltration of epithelium by polynuclear cells**	
0	No infiltration
1	Surface
2	Inside the crypt
3	Cryptic abscess
**Severity of epithelial damage**	
0	Absence of mucosal damage
1	Lymphoepithelial lesions
2	Mucosal erosion/ulceration
3	Extensive mucosal damage and extension throughout deeper structures of the bowel wall
**Surface of epithelial damage**	
0	Normal
1	Focal
2	Multifocal
3	Wide

The amount of recombinant mouse interleukin 1 beta, interleukin 6 and KC released in the culture supernatant of colonic tissue was determined by enzyme-linked immunosorbent assay (ELISA; R&D systems). The supernatant corresponded to colonic specimens (2 cm) incubated in DMEM/Ham's F12 medium supplemented with antibiotics (see paragraph on culture cells) for 24 H in an atmosphere containing 5% CO2 at 37°C.

### Statistical analysis/Phylogenetic analysis

Phylogenetic analysis of *fimH* and detection of adaptive amino acid changes in FimH were inferred using zonal phylogeny software [58]. Phylogenetic analysis of MLST data was based on allelic profiles and the minimum spanning tree was constructed using the Ridom Seqsphere software version 0.9 beta (Ridom GmbH, Münster, Germany). P-values were derived using 2×2 χ ^2^ statistics and p-values<0.05 were rated as significant. Quantitative data were compared by the Mann Whitney test.

### Ethics statement

This study was carried out in strict accordance with the recommendations of the Guide for the Care and Use of Laboratory Animals of the Université de of Clermont-Ferrand France. The animal protocol was approved by the Committee for Research and Ethical Issues of the Department of Auvergne (CEMEAAuvergne) following international directive 86/609/CEE (n°CE16-09). Informed written consent was obtained from all patients to isolate *E. coli* strains from biopsies or stools (CCPPRB Lille 1994 number 94/01 and CCPPRB Lille 2000 number 00/60).

## Supporting Information

Figure S1 Colony immunoblotting of AIEC LF82, LF82-Δ*fimH* and all the mutant LF82-Δ*fimH* expressing various *fimH* genes (/*fimH*_xx_) used in this study with type 1 pili antiserum.
(TIF)

Figure S2 Impact of FimH amino acid substitutions on the ability of AIEC to adhere to human intestinal T84 (A) and human urinary bladder T24 (B) cell lines after a 30 minute infection period.
(TIF)

Text S1 **Table S1**. Single-nucleotide polymorphism(s) for each fimH type. **Table S2**. Detailed phylogenetic information (phylotyping [ABD typing] and multilocus sequence typing [MLST]) of the 45 AIEC and 3 reference (MG1655 [E. coli K12], UPEC [CFT073], APEC) strains analyzed. In addition to the seven housekeeping genes analyzed (adk, fumC, gyrB, icd, mdh, purA, recA), the sequence type (ST) and ST complex are given. **Table S3**. Disease Activity Index (DAI) assessment.
(DOCX)
