# Supplementary material for: Point Mutations in FimH Adhesin of Crohn's Disease-Associated Adherent-Invasive Escherichia coli Enhance Intestinal Inflammatory Response
Source: PLoS Pathog. 2013 Jan 24;9(1):e1003141. doi: 10.1371/journal.ppat.1003141 (PMC3554634; doi:10.1371/journal.ppat.1003141)
Supplement: Text S1 — Table S1. Single-nucleotide polymorphism(s) for each fimH type. Table S2. Detailed phylogenetic information (phylotyping [ABD typing] and multilocus sequence typing [MLST]) of the 45 AIEC and 3 reference (MG1655 [E. coli K12], UPEC [CFT073], APEC) strains analyzed. In addition to the seven housekeeping genes analyzed (adk, fumC, gyrB, icd, mdh, purA, recA), the sequence type (ST) and ST complex are given. Table S3. Disease Activity Index (DAI) assessment. (DOCX) [file ppat.1003141.s003.docx]

**Legends for the Text S1 file**

**Table S1.** Single-nucleotide polymorphism(s) for each *fimH* type

**Table S2.** Detailed phylogenetic information (phylotyping [ABD typing] and multilocus sequence typing [MLST]) of the 45 AIEC and 3 reference (MG1655 [*E. coli* K12], UPEC [CFT073], APEC) strains analyzed. In addition to the seven housekeeping genes analyzed (*adk, fumC, gyrB, icd, mdh, purA, recA*), the sequence type (ST) and ST complex are given.

**Table S3.** Disease Activity Index (DAI) assessment

**Table S1.**

**Table S2.**

| Symptom/score | Characteristics |
| --- | --- |
| Body weight loss  0  1  2  3  4 | No loss  1-5% loss of body weight  5-10% loss of body weight  10-20% loss of body weight  >20% loss of body weight |
| Stool consistency  0  1  2  3  4 | Normal feces  Loose stool  Watery diarrhea  Slimy diarrhea, little blood  Severe watery diarrhea with blood |
| Blood in stool  0  2  4 | No blood  Presence of blood assessed by Hemoccult II test  Visible bleeding |

**Table S3.**
